# Supplementary material for: The effects of age and sex on cognitive impairment in schizophrenia: Findings from the Consortium on the Genetics of Schizophrenia (COGS) study
Source: PLoS One. 2020 May 13;15(5):e0232855. doi: 10.1371/journal.pone.0232855 (PMC7219730; doi:10.1371/journal.pone.0232855)
Supplement: S1 Methods — (DOCX) [file pone.0232855.s002.docx]

**Supplementary Method**

Participants

Before study initiation, interviewers at each site were trained to administer diagnostic interviews and clinical assessments using a standard training protocol. All participants were between 18 and 65 years old and received a modified version of the Structural Clinical Interview for DSM-IV (SCID) with additional items from the Diagnostic Interviews for Genetic Studies (DIGS) (Nurnberger et al., 1994) and the Family Interview for Genetic Studies (FIGS) (Maxwell, 1996). All patients met DSM-IV criteria for schizophrenia or schizoaffective disorder, depressed type. Controls were included if they had 1) no current or past psychotic disorder, 2) known biological family history, 3) no history of psychosis in 1st degree relative, 4) no current Axis I mood disorder, 4) no Cluster A Axis II disorder, and 5) no current regular treatment with psychoactive medication. Exclusion criteria for all participants were: 1) not being able to consent; 2) not fluent in English to understand the study procedure; 3) electroconvulsive therapy in the preceding 6 months; 4) positive drug toxicology screen for illicit drugs; 5) alcohol or substance abuse in the preceding 1 month; 6) alcohol or substance dependence in the preceding 6 months; 7) vision and hearing deficits that exclude more than 2 COGS-2 endophenotype measures; 8) head injury resulting in at least loss of consciousness > 15 minutes, personality change or onset of psychiatric symptoms after injury, residual neurological symptoms, abnormal neuroimaging or EEG findings attributed to the injury, or neurological illness – seizures, cerebrovascular accident, Parkinson’s Disease; 9) severe systemic illness (i.e., congestive heart failure, insulin-dependent diabetes mellitus Type 1 and Type 2) that interferes with ability to be tested; 10) premorbid IQ less than 70 as determined by the Wide Range Achievement Test-3 (WRAT-3) (Wilkinson, 1993); 11) previous neuropsychological testing in the preceding 3 months; 12) previous endophenotype testing in the preceding 1 month; 13) being pregnant; and 14) first-degree relative who has already participated in this study.
